# Regulation of TFEB activity and its potential as a therapeutic target against kidney diseases

**DOI:** 10.1038/s41420-020-0265-4

**Published:** 2020-05-01

**Authors:** Weihuang Zhang, Xiaoyu Li, Shujun Wang, Yanse Chen, Huafeng Liu

**Affiliations:** Institute of Nephrology, and Key Laboratory of Prevention and Management of Chronic Kidney Disease of Zhanjiang City, Affiliated Hospital of Guangdong Medical University, 524001 Zhanjiang, Guangdong China

**Keywords:** Nephrons, Acute kidney injury

## Abstract

The transcription factor EB (TFEB) regulates the expression of target genes bearing the Coordinated Lysosomal Expression and Regulation (CLEAR) motif, thereby modulating autophagy and lysosomal biogenesis. Furthermore, TFEB can bind to the promoter of autophagy-associated genes and induce the formation of autophagosomes, autophagosome–lysosome fusion, and lysosomal cargo degradation. An increasing number of studies have shown that TFEB stimulates the intracellular clearance of pathogenic factors by enhancing autophagy and lysosomal function in multiple kidney diseases, such as cystinosis, acute kidney injury, and diabetic nephropathy. Taken together, this highlights the importance of developing novel therapeutic strategies against kidney diseases based on TFEB regulation. In this review, we present an overview of the current data on TFEB and its implication in kidney disease.

## Facts

- The transcription factor EB regulates the expression of CLEAR motif-containing target genes involved in autophagy and lysosomal biogenesis.
- Aberrant autophagy and impaired lysosomal function are important in kidney diseases.
- TFEB deficiency is involved in the development of kidney diseases.
- Thus, targeting TFEB activity, autophagy, and mitophagy could be a novel therapeutic strategy for patients with kidney diseases.

## Introduction

Autophagy is an evolutionarily conserved intracellular homoeostatic process wherein cytoplasmic cargo-containing autophagosomes fuse with lysosomes to degrade the cargo^1^. Macroautophagy and selective autophagy (e.g., mitophagy, aggrephagy) influence cellular processes, such as cell death, inflammation, and immune responses, thereby exerting adaptive and maladaptive roles in the pathogenesis of multiple human diseases, such as skeletal muscle diseases, cancer, neurodegenerative diseases, systemic lupus erythematosus, chronic kidney disease etc.^2,3^.

Transcription factor EB (TFEB), a basic helix–loop–helix-leucine-zipper (bHLH-Zip) protein in the microphthalmia/transcription factor E (MiT/TFE) family, primarily controls the expression of genes in the autophagy–lysosomal pathway^4–6^. TFEB regulates autophagic flux by promoting the biogenesis of lysosomes, formation of autophagosomes, and fusion with lysosomes, thereby facilitating substrate clearance. TFEB also functions in selective autophagy and lysosomal exocytosis^7^. Overexpression of TFEB enhances the degradation of bulk amounts of substrates, lipid droplets, and damaged mitochondria and alleviates the phenotypes associated with various diseases, such as Parkinson’s and Alzheimer’s disease, in murine models by promoting autophagy and lysosomal biogenesis^8^. In particular, current researches declared that TFEB is associated with kidney disease pathogenesis in diverse conditions, such as diabetic nephropathy (DN)^9^ and acute kidney disease^10^. In this review, we have described the mechanisms involved in the regulation of TFEB activation and, subsequently, coordinating lysosomal function and autophagy. We have emphasised the role of TFEB in kidney diseases and its potential as a therapeutic in rescuing renal function.

## MIT/TFE family of transcription factors

**Fig. 1:**
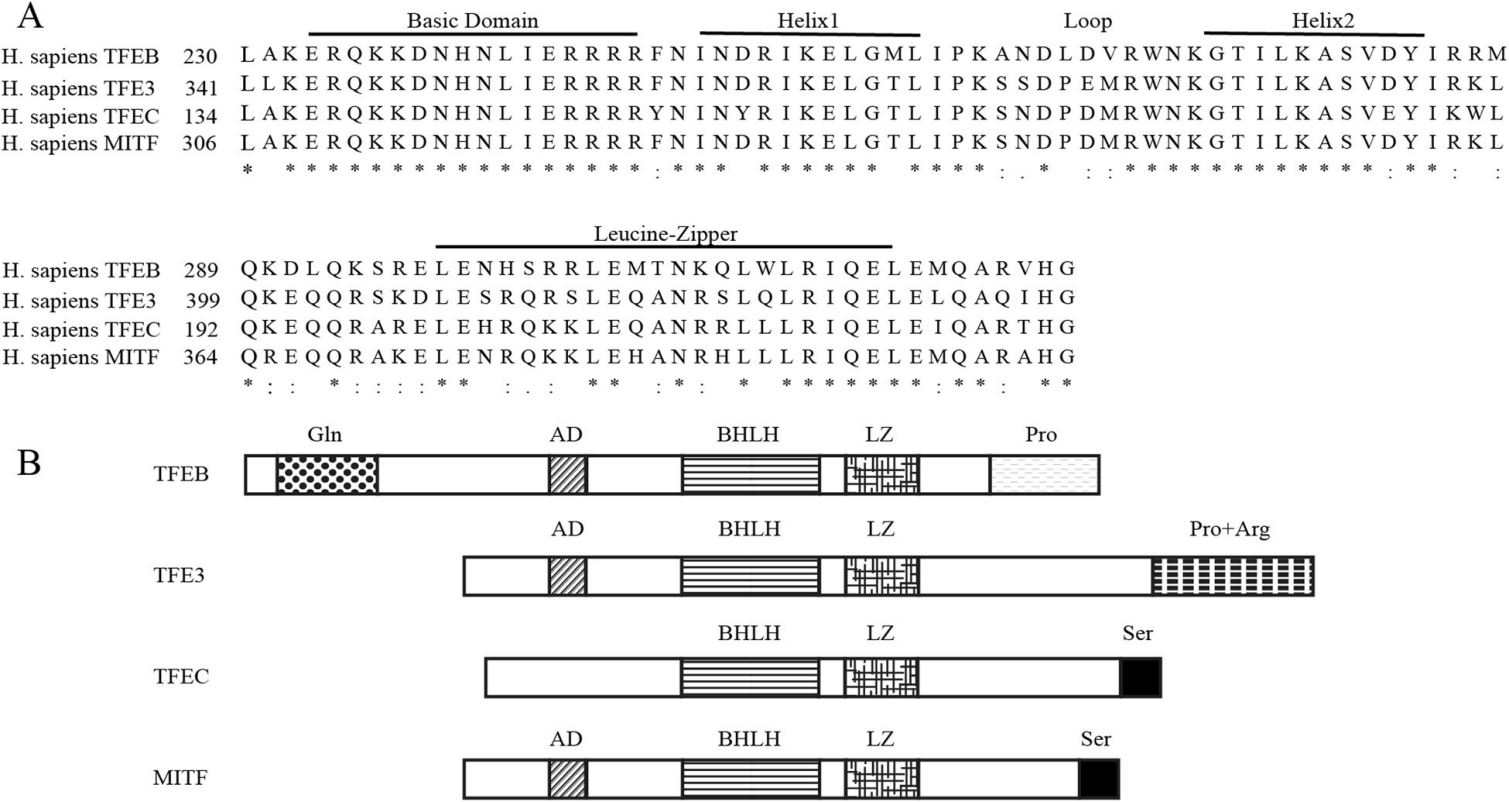
The four members of MIT/TFE family of proteins. **a** Comparison of the amino acid sequences of the bHLH and leucine zipper regions of TFEB, TFE3, TFEC, and MITF. **b** The various domains found in TFEB, TFE3, TFEC, and MITF. Gln glutamine-rich region, AD acidic domain, bHLH basic helix–loop–helix, LZ leucine zipper domain, Pro proline-rich segment, Pro+Arg proline- and arginine-rich region, Ser serine-rich region.

**Fig. 2:**
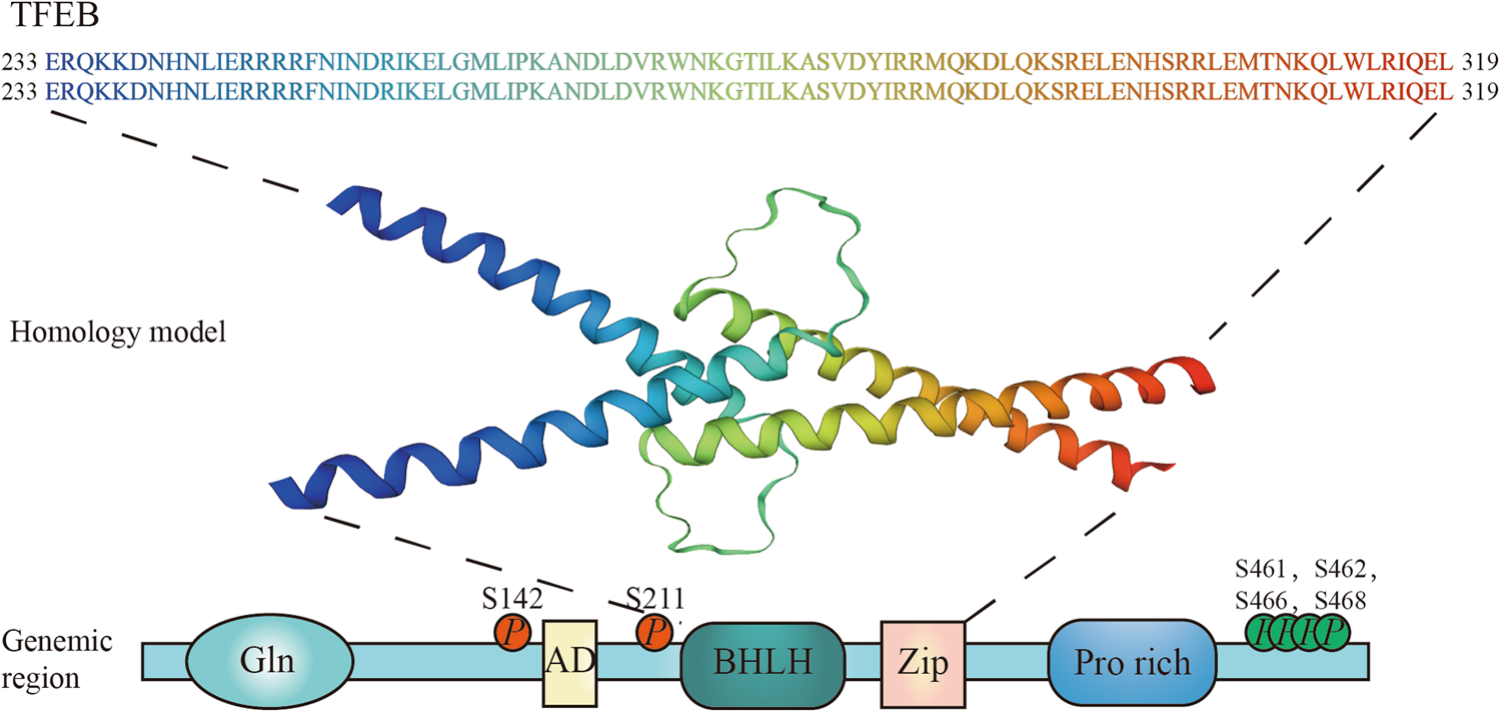
Domain structure and homology model of TFEB. The homology model was adopted from the SWISS-MODEL (https://swissmodel.expasy.org/; a fully automated protein structure homology-modelling server).

Four members of the microphthalmia family of bHLH-Zip transcription factors have been identified: MITF/TFEF, TFEB, TFE3, and TFEC^4,8^ (Fig. 1). The common features of the MIT/TFE proteins include a DNA-binding motif, HLH, and Zip region necessary for dimerisation^4^. MITF/TFEF, TFEB, and TFE3 also possess an activation domain required for its transcriptional activation function^8,11^. TFEC does not contain this activation domain and seems to play a role of inhibition to its downstream gene’s transcription rather than activation^12^ (Fig. 2 shows the domain structure and homology model of TFEB.)

MIT/TFE proteins are conservative in vertebrates^13^ and primarily expressed in the retinal pigment epithelia, macrophages, osteoclasts, mast cells, melanocytes, and natural killer cells^14^, while TFEC expression is limited to myeloid cells^15^. TFE3 and TFEB are expressed in multiple cell types^16^. Researches of MIT/TFE proteins have shown that they play a critical role in the maintenance of physiological and pathological processes^17^. Aberrant expression of these proteins stimulate the development of various human cancers, including renal carcinomas^18,19^, melanomas^20^, and alveolar sarcomas^21^.

## Transcriptional control in lysosomes by TFEB

The lysosome was discovered in the early 1950s as a membrane-bound organelle containing more than 50 types of acid hydrolases for a wide variety of substrates, including proteins, carbohydrates, lipids, and nucleic acids^22^. Lysosomes are primary sites of intracellular degradation and molecular recycling system and maintenance of cellular homoeostasis^23^. Lysosomes are crucial for endocytosis, autophagy, and lysosomal exocytosis^24^. Lysosomal genes share a 10-base E-box-like palindromic sequence (GTCACGTGAC) typically found within 200 base pairs of the transcription initiation site. This motif, named the Coordinated Lysosomal Expression and Regulation (CLEAR) element, comprises an E-box (CANNTG) that was recognised by the MIT/TFE family transcription factors^5^.

TFEB enhances the expression of its target genes by specifically binding to the CLEAR motif present in the target promoters^5,7^. Thus, TFEB overexpression increases the biogenesis of lysosomes and improves their capacity for degrading lysosomal substrates, such as glycosaminoglycans and substrates for autophagy^5,8^. In addition, TFEB can promote lysosomal exocytosis which is a process that lysosomes can secrete content out of cell through fusing to cell membrane^25^. This highlights the importance of transcriptional control of gene expression in lysosomal function.

**Fig. 3:**
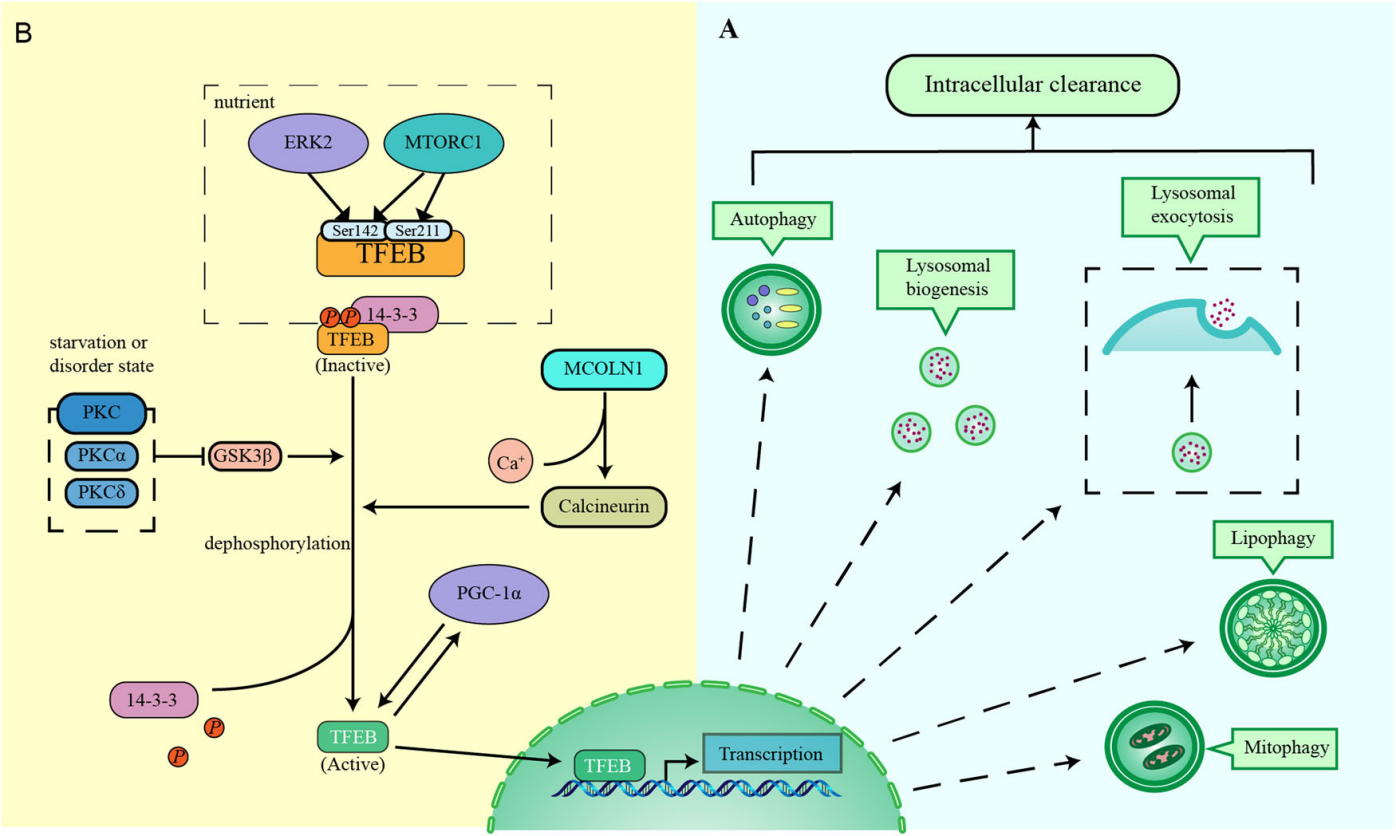
TFEB function and mechanism of TFEB activation. **a** TFEB regulates several biological processes, such as autophagy, lysosomal biogenesis, lysosomal exocytosis, lipophagy, mitophagy, etc. **b** Under nutrient-rich conditions, TFEB is predominantly found in its inactive state that is characterised by mTORC1 and ERK2-mediated phosphorylation. When cells experience stress or disease, TFEB is activated by dephosphorylation.

TFEB is involved in intracellular clearance by enhancing lysosomal biogenesis and function, autophagy, and lysosomal exocytosis (Fig. 3a)^7^. TFEB overexpression improves the rate of degradation of the cargo, e.g., long-lived proteins^6^ and enhances the elimination of lipid droplets^26^ and damaged mitochondria^27^. Hence, TFEB is the central modulator of the autophagy–lysosomal pathway and organelle-specific autophagy, including lipophagy and mitophagy.

## Mechanism involved in regulating TFEB activity

TFEB regulates biological processes by modulating the expression of downstream genes. TFEB activity is tightly controlled by post-translational modifications and protein–protein interactions^8^. During nutrient-rich conditions, TFEB is predominantly in its inactive phosphorylated form in the cytosol^5,6^. However, under conditions of starvation, oxidative stress, or lysosomal disorder, TFEB translocates to the nucleus to bind its target genes and stimulate autophagy and lysosomal function^28^ (Fig. 3b).

The phosphorylation state of TFEB depends on two serine residues (Ser142 (refs. ^6,29^) and Ser211 (refs. ^30,31^)) that regulates its activity and cellular localisation. When Ser142 and Ser211 are phosphorylated, TFEB is inactive and localises to the cytoplasm. Mutating either of the serine residues to alanine enables TFEB to be constitutively active and localise to the nucleus^6,29–31^.

Mechanistic target of rapamycin complex 1 (mTORC1) is an atypical Ser/Thr kinase that is stimulated by the nutrient content and provides balance between anabolism and catabolism^32^. Ser211 is phosphorylated by mTORC1, which phosphorylates TFEB at Ser211 and induces the binding between TFEB and 14-3-3 proteins, thereby inhibiting TFEB from shuttling into the nucleus. After mTORC1 is inhibited, TFEB gets dephosphorylated and translocates into the nucleus^30,31,33^. It has been recently reported that mTORC1 can phosphorylate Ser211 and Ser122 to regulate TFEB activity^32^. Ser142, another serine involved in the subcellular localisation of TFEB, is phosphorylated by mTORC1 and ERK2 (refs. ^6,29^). Studies have shown that TFEB phosphorylation at Ser142 competes with that at Ser211, but the mechanism is yet to be elucidated^34,35^. Therefore, understanding the phosphorylation status of these amino acid residues is critical for TFEB activity and localisation.

ERK2 is another important Ser/Thr kinase that regulates cell proliferation, differentiation, transformation, apoptosis, and autophagy^6,36^. Under normal cellular conditions, ERK2 phosphorylates Ser142 in TFEB and holds its cytosolic localisation. During cell starvation, ERK2 no longer maintains the phosphorylation of TFEB, thereby activating TFEB and enabling its translocation to the nucleus^6^. ERK2 localises to multiple subcellular compartments, including the lysosome^37^. Further research is required to understand whether ERK2-mediated TFEB phosphorylation occurs on the lysosomal surface or in the other subcellular compartments.

Calcineurin is a serine/threonine phosphatase that regulates TFEB activity^38^. During nutrient deficiency or lysosomal stress, Ca^2+^ is released from the lysosome via mucolipin 1, thereby stimulating calcineurin, TFEB dephosphorylation, and nuclear translocation^33^. Upon nutrient deprivation, the depletion of mucolipin 1 decreases the activation of TFEB and inhibits autophagy by hindering lysosomal Ca^2+^ release and calcineurin activation^33^.

The protein kinase C (PKC) family of proteins comprise Ser/Thr kinases that are involved in cell growth, differentiation, apoptosis, transformation, tumorigenicity, synaptic function, behaviour, and cognition^39^. Activated isoforms of PKC, primarily PKCα, PKCβ, and PKCδ, stimulate the biogenesis of lysosomes by coupling two parallel signalling pathways without affecting mTORC1. Activated PKCα and PKCδ inactivate GSK3β, a ubiquitous Ser/Thr kinase that is involved in translational regulation, thereby activating TFEB via reduced phosphorylation and increased nuclear localisation^35^. PKCδ phosphorylates JNK and p38 MAPK, thereby inactivating ZKSCAN3 and translocating it out of the nucleus^35,40^. Therefore, PKC activates TFEB and inactivates the transcriptional repressor ZKSCAN3 by targeting two parallel signalling cascades.

Interestingly, some regulators of TFEB are in turn regulated by TFEB. PGC-1α (peroxisome proliferator-activated receptor g coactivator 1 α) is the main modulator of mitochondrial biogenesis and respiration^41,42^. Several reports show that TFEB regulates mitochondrial biogenesis and glucose through controlling cellular uptake of PGC-1α^26,42^. Moreover, PGC-1α upregulates and activates TFEB^26^. These phenotypes demonstrate that the regulation of and by TFEB dictates survival in response to environmental stresses that is balanced by numerous feedback loops^8^. Furthermore, TFEB upregulation induces its own transcriptional activation^26^, revealing the presence of another feedback loop that controls lysosomal biogenesis and function.

## Research status and application of regulated TFEB activity as therapy in kidney diseases

### Cystinosis

Cystinosis is a lysosomal storage disorder due to mutations in CTNS gene (encoding cystinosin) that is a proton-cystine symporter responsible for the secretion of cystine dimers and protons from the lysosomal lumen^43–45^. Cystinosis is a rare autosomal recessive disease that is found in 1 out of 100,000 individuals^46^. Cystinosis manifests with the accumulation of cystine in lysosomes, thereby resulting in cellular dysfunction and affecting multiple organs, such as kidneys, eyes, liver, and brain^47^. Fanconi syndrome occurs during the first year of life and is characterised by kidney (primarily proximal tubular) dysfunction with polyuria and loss of glucose, proteins, and amino acids in the urine^47,48^. Without treatment, patients develop progressive glomerular damage and end-stage renal disease (ESRD) in ~10 years^49^. ESRD, dehydration, and electrolyte imbalance can be attributed to the rate of mortality in patients with Fanconi syndrome^46^.

It has been recently reported that proximal tubular injury results in increased incidence of apoptosis^50^, anomalous autophagy^51,52^, endoplasmic reticulum stress^53^, endo-lysosomal dysfunction^54^, and cell dedifferentiation^55^. Cysteamine has been the only treatment available for cystinosis since the late 1970s^48,56^; it reduces lysosomal cystine content and delays glomerular aggravation while improving growth of children with cystinosis. However, even treated with cysteamine, patients require a kidney transplantation owing to cellular dysfunction, progressive renal injury, and tissue failure^49,57,58^. Thus, there is an urgent need for developing novel therapeutic strategies against cystinosis that improves the quality of life and prognosis of patients with nephropathic cystinosis.

TFEB is the primary transcription factor involved in regulating the expression of lysosomal elements, including cystinosin, and lysosomal biogenesis and clearance by coordinating autophagy and anabolism^24^. Similar to the lysosome-related genes, TFEB regulates the expression of CTNS by binding to the E-box in its promoter^7^. Furthermore, Rega et al.^48^ reported that cystinotic cells, derived from a patient with cystinosis or CTNS-depleted (short interfering RNA-mediated) healthy cells, possess decreased levels of TFEB that cannot be rescued by cysteamine. Moreover, depleting cells of CTNS results in the decrease of TFEB without affecting the accumulation of intracellular cystine, indicating that decreased levels of TFEB lead to the deficiency in cystinosin, but not cystine accumulation. Similarly, Zhang et al.^58^ demonstrated that CTNS^−^^/−^ mouse fibroblasts exhibited downregulated endogenous TFEB that, upon upregulation, promoted the expression of cystinosin and rescued cystinosis. Andrzejewska et al.^59^ showed that clearance of lysosomal cystine using cysteamine cannot reactivate mTORC1 (upstream effector of TFEB) in cystinotic cells, further suggesting that mTORC1 inactivation is a result of cystinosin deficiency rather than cystine accumulation and cystinosin directly regulates mTORC1 activity. Thus, cystinosin functions directly, and indirectly, in regulating the intracellular expression and activity of TFEB, thereby limiting cysteamine from fully curing patients with Fanconi syndrome.

Cystinosis is also characterised by the presence of abnormal lysosomes that result in aberrant autophagy. Ivanova et al.^54^ showed that cells deficient for cystinosin have atypical shape, disordered endo-lysosomal compartments, and damaged endocytosis. Cysteamine treatment promotes surface expression of receptors (megalin and cubilin) and elimination of lysosomal substrates but failed to recover the aberrant morphology of the endo-lysosomal compartments. Activation of TFEB increases its expression, nuclear translocation, and induction of lysosome biogenesis by genistein, thereby effectively promoting the clearance of aggregates^60–63^. Impaired autophagy has been recently shown to be associated with the pathogenesis of cystinosis^52,64^. Genetic and chemical manipulation stimulates TFEB activity that promotes the clearance of accumulated cystine, degradation of delayed cargo, and recovery of the aberrant morphology of the lysosomal compartments in cystinotic cells^48^. Taken together, cystinosis results from the reduction in expression and activity of TFEB and regulating TFEB may be a potential therapeutic strategy against cystinosis.

### Acute kidney injury

Acute kidney injury (AKI) is a commonly occurring disease of the kidney that affects ∼13.3 million patients and causes 1.7 million deaths per year^65^ and characterised by sudden kidney failure or injury, thereby resulting in the deposition of metabolic end-products and/or decreased urine output^66^. Patients with AKI can develop chronic kidney disease and are at a higher risk of ESRD in the future^67^. The pathogenesis of AKI involves inflammation, damage to the tubular epithelia, endothelial and vascular disorder, reperfusion, sepsis, and nephrotoxins^68–73^. The renal tubules are severely injured in patients with AKI that results in tubular malfunction and necrosis and apoptosis; proximal tubules are particularly vulnerable to injury^70–75^. Tubular cells that survive restore the integrity of the remnant tubule by increased proliferation; inaccurate and suboptimal repairs lead to renal fibrosis and, ultimately, chronic kidney disease^76^.

Autophagosomes were discovered in tubular epithelial cells from a rodent model for renal ischaemia–reperfusion injury (IRI)^77^. Subsequently, the induction of autophagy has been observed in in vivo and in vitro models of IRI. Inhibiting autophagy aggravates IRI as indicated by renal function, histology, and tubular cell apoptosis, suggesting that autophagy plays a renoprotective role in cell survival in IRI^78^. Cisplatin-induced AKI is similar to IRI: inhibiting autophagy using pharmacological inhibitors (3-methyladenine or bafilomycin A1) or genetic manipulation (knockdown of Beclin-1 or ATG5) increases apoptosis in cisplatin-treated RPTC cells, suggesting autophagy is important in protecting renal function in cisplatin-induced tubular cell injury^79^. The group of Yang et al.^80^ showed that cisplatin-induced autophagy in LLC-PK1 cells play a protective role against cell apoptosis. Similarly, cisplatin-treated AKI model revealed that the inhibition in autophagic flux remarkably enhances kidney injury, while the activation of autophagy protects proximal tubules from injury^81^. Wang et al.^10^ demonstrated that TFEB, activated by urolithin A, promotes autophagy by regulating CLEAR motif-containing genes and attenuates IRI by reducing inflammation and kidney injury. Thus, the induction of autophagy by TFEB can be used as a novel therapeutic strategy for AKI.

Mitochondria are dynamic organelles that undergo continuous biogenesis, fusion, and fission and are selectively removed by mitophagy; all of these processes are crucial for its function and have been proven to be associated with AKI^82^. Whitaker et al.^83^ demonstrated disrupted mitochondrial homoeostasis and biogenesis after ischaemia–reperfusion, rhabdomyolysis-induced AKI, and folic acid-induced AKI. Tran et al.^84^ indicated inhibiting PGC-1α and mitochondrial biogenesis may take part in disease progression in sepsis-induced AKI. Persistent stimulation of mitochondrial biogenesis continually replenishes the cellular mitochondrial content and ameliorates tubular damage induced by ischaemia/reperfusion, toxins, and oxidants^85,86^. Disrupted mitochondrial dynamics result in mitochondrial dysfunction, tubular cell damage, and death in ischaemia–reperfusion and cisplatin-induced AKI^86^. Gonzalez et al.^87^ demonstrated that the imbalance between mitochondrial fusion and fission determines the differential progression of experimental sepsis. Liu et al.^88^ found that the balance between mitochondrial fusion/fission is tilted towards fission in the later stages of sepsis-induced AKI. They also reported impaired mitophagy during this stage although mitophagy is stimulated in the earlier stages of disease. Mitophagy maintains mitochondrial biogenesis and energy metabolism by removing dysfunctional or excrescent mitochondria and preventing the accumulation of reactive oxygen species, inflammation, and apoptosis^89–92^. Therefore, moderating mitochondrial biogenesis, mitochondrial dynamics, and mitophagy may be beneficial in preventing AKI. As Scott et al*.*^93^ have shown that TFEB (downstream effector of PGC-1α) controls the cellular mitochondrial content. Lynch et al.^94^ demonstrated that TFEB is required for PGC-1α-dependent tubular protection because PGC-1α promotes lysosomal biogenesis via TFEB as AKI progressing. Tan et al.^95^ reported that pomegranate extract potentiates mitophagy to eliminate the accumulation of dysfunctional mitochondria and reactive oxygen species in a TFEB-dependent manner. Liu et al.^96^ demonstrated that TFEB overexpression induces mitophagy by promoting the expression of autophagy-related genes. Moreover, Prajapati et al.^97^ found that TFEB expression induces the biogenesis of lysosomes and promotes the recovery of mitochondrial function. Taken together, TFEB activation promotes mitophagy and rescues mitochondrial function and can be a promising candidate to target AKI.

As mentioned, TFEB plays a critical role in promoting lysosomal function and autophagy by regulating the CLEAR motif-containing genes. TFEB and the CLEAR network are associated with molecular interactions in AKI. TFEB binds to the promoters of its targets and regulates various degradation pathways, such as glycosaminoglycan, haemoglobin, chitin, and sphingolipid^7^, that are critical in AKI^98,99^. TFEB also regulates other processes associated with AKI^100–102^, such as protein degradation, energy metabolism, steroid biosynthesis, antigen processing and presentation, signalling pathways, and DNA metabolism^7^. TFEB is involved in eliminating dead and injured cells (critical for the recovery of AKI^7^). Taken together, TFEB is a promising therapeutic target for AKI.

### Diabetic nephropathy

DN is a serious complication associated with diabetes and one of the most important factors in the development of ESRD worldwide^103^. Approximately 35–40% diabetic patients (type 1 or 2) ultimately develop DN; this has led to the poor clinical outcome and increase in mortality in diabetic patients^104^. The International Diabetes Federation has predicted a global increase in the number of patients with DN from 382 million to 592 million between 2013 and 2035 (ref. ^105^). Unfortunately, this will be associated with increased economic burden for patients and the society.

Diabetic patients manifest with disrupted multi-factor interactions between metabolic processes and haemodynamics, thereby enabling the development of DN^104^. Clinical studies have shown that hyperglycaemia is the primary risk factor for complications associated with diabetes, including DN^106^. DN can be attributed to the alterations in cellular metabolism mediated by hyperglycaemia, including the accumulation of advanced glycation end-products (AGEs), activation of PKC, and oxidative stress^107,108^. Furthermore, chronic hyperglycaemia activates the diacylglycerol–PKC pathway that regulates vascular permeability, vasoconstriction, and the synthesis and turnover of the extracellular matrix^109^. Moreover, the pathogenesis of DN is influenced by the haemodynamic changes that lead to systemic and glomerular hypertension and renin–angiotensin system^110–113^.

Impaired autophagy has also been reported to be involved in the pathogenesis of DN. Renal cortical tubules of streptozotocin (STZ)-induced early diabetic rats exhibit inhibited autophagy that can be reversed upon treatment with insulin or islet transplantation^114,115^. Impaired autophagy results in the renal deposition of p62/Sequestosome 1 (SQSTM1) and degradation products from autophagy in mouse models of type 1 and type 2 diabetes, respectively^116,117^. Liu et al.^118^ showed that autophagosomes and SQSTM1-positive amass in tubular epithelial cells in patients with DN or HK-2 cells treated with AGEs, indicating that AGEs disturb autophagy–lysosomal pathway of renal tubular epithelial cell. Moreover, biopsies from animal models and human kidney showed that aberrant autophagy is involved in renal cell injuries and the pathogenesis of DN^119^.

Recent studies have revealed that impaired autophagy contributes to the development of DN via nutrient-sensing pathways. Excess nutrition leads to the activation of mTOR and reduction of AMPK (AMP-activated protein kinase) and Sirt1 (Sirtuin 1), thereby leading to the inhibition of autophagy in human and experimental type 1 and 2 DN^120–123^. Autophagy can be inhibited in podocytes upon prolonged treatment with high concentrations of glucose that can be recovered by the addition of rapamycin, indicating that mTOR inhibits autophagy during hyperglycaemia, leading to podocyte injury^124^. Similarly, impaired autophagy can be reversed by rapamycin-mediated inhibition of mTOR in STZ-induced diabetic mice^125^. Zhao et al.^126^ demonstrated that mTOR-induced autophagic dysfunction can be attributed to podocyte injury stimulated by high content of AGEs; pharmacologic downregulation of mTOR reverses impaired autophagy. This reinforces the potential for targeting autophagic flux in patients with DN. Thus, TFEB regulates autophagy via the mTOR pathways and, as compared to mTOR, is a more direct target to activate autophagy.

DN is also characterised by TFEB dysfunction. Brijmohan et al.^9^ found that in humans with DN, the accumulation of misfolded proteins coincided with a decrease in TFEB expression. Takahashi et al.^127^ demonstrated that lysosomal biogenesis and function were induced in vitro by increasing amounts of AGEs, but was inhibited in autophagy-deficient renal proximal tubular epithelial cells due to the cytoplasmic retention of TFEB. Similarly, STZ-treated mice with defective autophagy do not induce lysosomal biogenesis and exhibit amassed AGEs in the glomeruli, renal vasculature, and proximal tubular epithelial cells. These findings indicate that autophagy promotes the clearance of AGEs by increasing lysosomal biogenesis and function; this can be augmented by activating TFEB. Zhao et al.^126^ also showed that AGEs inhibit the formation and turnover of autophagosomes in podocytes by disrupting the binding of TFEB to the promoters of autophagy-related genes and activating mTOR to prevent the transcriptional activation of TFEB. Conversely, TFEB overexpression restores autophagic flux, confirming that downregulation of TFEB leads to impaired autophagy stimulated by AGEs in podocytes. Taken together, these studies reveal a pivotal role of TFEB in the pathogenesis of DN that can be used as a therapeutic strategy to protect autophagy.

## Conclusion

TFEB is an important transcriptional modulator that regulates the expression of genes involved in the autophagy–lysosomal pathways, thereby controlling intracellular clearance and energy metabolism. Understanding the role of TFEB has helped provide insights into the mechanisms involved in cellular response to environmental conditions, such as nutrient deficiency. The advancements in research on TFEB and MIT/TFE proteins have led to a better understanding of the function, regulatory mechanisms, and pathways affected by these transcription factors. However, how cells integrate multiple extra- and intracellular signals to modulate an appropriate response remains to be investigated. Numerous studies have shown that improving intracellular clearance may alleviate the symptoms associated with a variety of diseases, especially neurodegenerative diseases. Regulating TFEB activity may be a promising therapeutic strategy against kidney diseases (Table 1). However, the function and mechanism of TFEB involved in the treatment process of kidney disease are still notemployed by TFEB in these processes remain to be fully understood. Therefore, further studies need to be performed to develop TFEB as a therapeutic target for kidney diseases.

**Table 1:** Presumed therapeutic mechanism of TFEB activation in kidney diseases.

Therapeutic strategies	Kidney diseases	Presumed mechanism of action	Main reference
Activation or overexpression of TFEB	Cystinosis	Upregulates cystinosin	^5,48,54,58^
		Rescues the aberrant lysosomes	
		Enhances autophagy	
	Acute kidney injury	Enhances autophagy	^10,78,79,82,94^^,^^95^
		Enhances mitochondrial biogenesis	
		Enhances mitophagy	
		Improves mitochondria function	
	Diabetic nephropathy	Improves impaired autophagic activity	^9,118,119,126,127^
		Recovers amount and function of TFEB	
		Reduces AGEs accumulation	
